# The involvement of brain regions associated with lower KPS and shorter survival time predicts a poor prognosis in glioma

**DOI:** 10.3389/fneur.2023.1264322

**Published:** 2023-12-04

**Authors:** Hongbo Bao, Huan Wang, Qian Sun, Yujie Wang, Hui Liu, Peng Liang, Zhonghua Lv

**Affiliations:** ^1^Department of Neurosurgery, Harbin Medical University Cancer Hospital, Harbin, Heilongjiang, China; ^2^Department of Neurosurgery, Beijing Tiantan Hospital, Capital Medical University, Beijing, China

**Keywords:** GBM, astrocytoma, IDH, VLSM, survival time, KPS

## Abstract

**Background:**

Isocitrate dehydrogenase-wildtype glioblastoma (IDH-wildtype GBM) and IDH-mutant astrocytoma have distinct biological behaviors and clinical outcomes. The location of brain tumors is closely associated not only with clinical symptoms and prognosis but also with key molecular alterations such as IDH. Therefore, we hypothesize that the key brain regions influencing the prognosis of glioblastoma and astrocytoma are likely to differ. This study aims to (1) identify specific regions that are associated with the Karnofsky Performance Scale (KPS) or overall survival (OS) in IDH-wildtype GBM and IDH-mutant astrocytoma and (2) test whether the involvement of these regions could act as a prognostic indicator.

**Methods:**

A total of 111 patients with IDH-wildtype GBM and 78 patients with IDH-mutant astrocytoma from the Cancer Imaging Archive database were included in the study. Voxel-based lesion-symptom mapping (VLSM) was used to identify key brain areas for lower KPS and shorter OS. Next, we analyzed the structural and cognitive dysfunction associated with these regions. The survival analysis was carried out using Kaplan–Meier survival curves. Another 72 GBM patients and 48 astrocytoma patients from Harbin Medical University Cancer Hospital were used as a validation cohort.

**Results:**

Tumors located in the insular cortex, parahippocampal gyrus, and middle and superior temporal gyrus of the left hemisphere tended to lead to lower KPS and shorter OS in IDH-wildtype GBM. The regions that were significantly correlated with lower KPS in IDH-mutant astrocytoma included the subcallosal cortex and cingulate gyrus. These regions were associated with diverse structural and cognitive impairments. The involvement of these regions was an independent predictor for shorter survival in both GBM and astrocytoma.

**Conclusion:**

This study identified the specific regions that were significantly associated with OS or KPS in glioma. The results may help neurosurgeons evaluate patient survival before surgery and understand the pathogenic mechanisms of glioma in depth.

## 1 Introduction

Gliomas, originating from glial cells, are the most common type of malignant brain tumors in adults (1). According to the fifth edition of the WHO Classification of Tumors of the Central Nervous System (WHO CNS5), adult-type diffuse gliomas can be divided into isocitrate dehydrogenase-wildtype glioblastoma (IDH-wildtype GBM), IDH-mutant astrocytoma, and IDH-mutant and 1p/19q-codeleted oligodendroglioma (2). Compared with IDH-mutant astrocytoma and oligodendroglioma, IDH-wildtype GBM is more aggressive, with a median survival of 12–16 months (3, 4). Despite advances in novel therapies, the outcome of glioma remains dismal. Therefore, a more comprehensive understanding of factors that influence prognosis is necessary.

Accumulating evidence suggests that the anatomic location is one of the factors that may affect tumor progression and clinical outcomes. For example, glioma's synchronous invasion into the corpus callosum and the subventricular zone has been proven to be a significant adverse predictor of radiotherapy sensitivity, overall survival (OS), and progression-free survival (PFS) (5, 6). Deep GBM location and location within eloquent brain regions, such as the motor/sensory cortex, internal capsule, basal ganglia, thalamus, and brainstem, are more likely to be related to functional disorders and neurologic deficits, such as seizures, motor or sensory deficits, and aphasia (7, 8). Even small tumors in these eloquent regions can induce obvious symptoms and raise clinical concerns earlier than those with similar volumes in non-eloquent areas. Recently, studies have also demonstrated that the localization of glioma is associated with certain characteristics of patients, including age (9), sex (10), tumor molecular alterations (11–14), and clinical presentation (7). When patients are stratified by sex, GBM lesions are more likely to localize to the left temporal lobe in men and involve more right temporal lobe in women. Voxel-based lesion-symptom mapping (VLSM), as a statistical technique, allows for the generation of eloquence maps that quantify the discrepancy in each voxel between patients with and without a lesion regarding a clinical outcome score (15). VLSM analysis has identified that the anterior horn of the lateral ventricles is one of the high-probability locations for IDH-mutant tumors in both GBM and low-grade glioma (LGG) (14). However, how tumor location affects patient prognosis and how neurosurgeons make clinical decisions according to location remain largely unclear.

The Karnofsky Performance Scale (KPS), a simple and reliable tool to assess patients' functional performance, has been widely used in medical oncology. In a nomogram based on multivariable analysis, the KPS score was found to be the largest contributor to OS, followed by chemotherapy, IDH mutation status, and surgical resection (16). Moreover, it has been shown that the KPS score is a promising prognostic indicator in both primary and recurrent glioma patients (17, 18).

**Figure 1:**
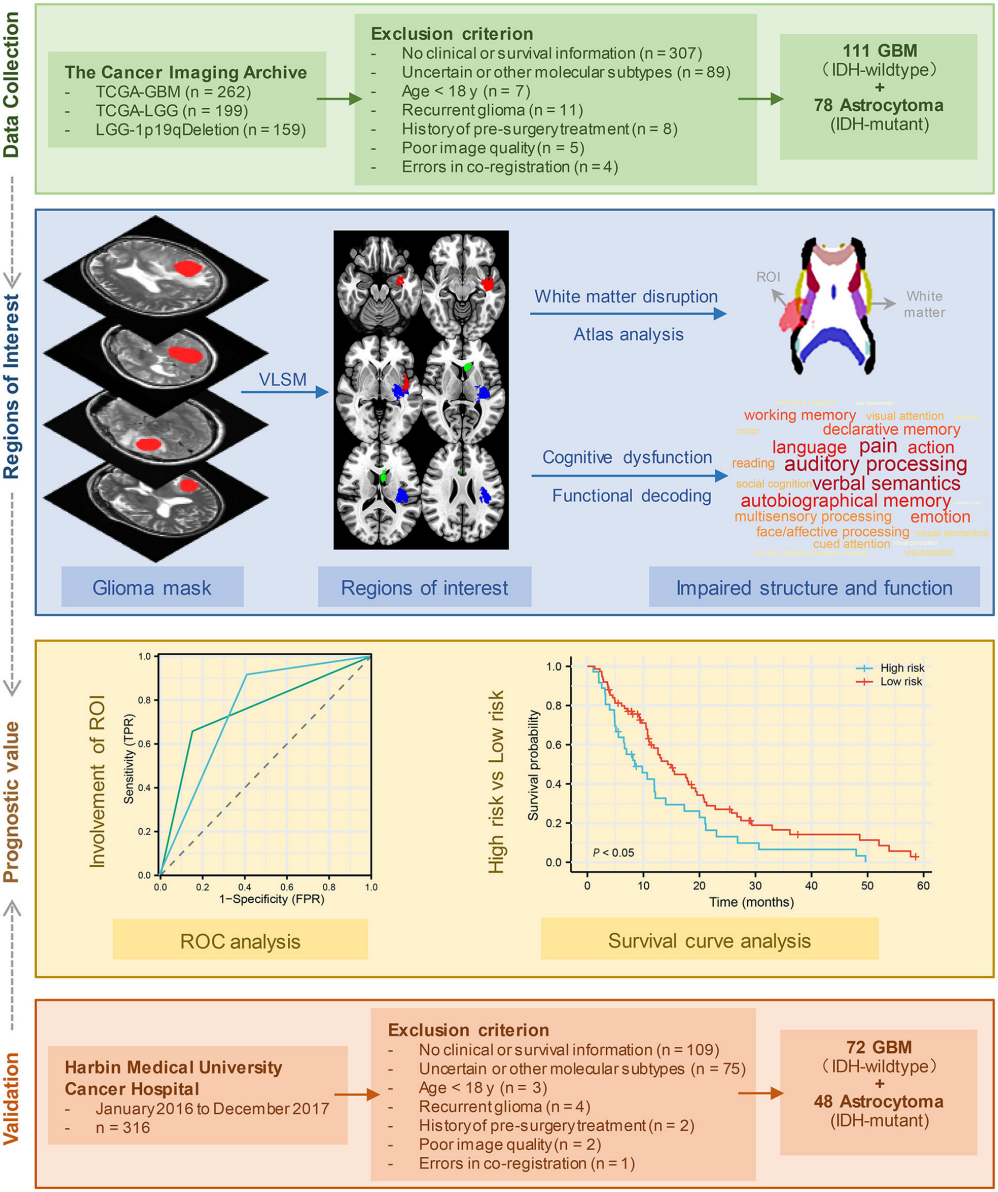
Flow diagram of study design.

The present study attempts to offer an in-depth understanding of the factors that impact survival time and KPS (Figure 1). We started by identifying brain regions that were associated with the KPS score and OS in IDH-wildtype GBM and IDH-mutant astrocytoma separately. Next, we explored the structural and functional impairments associated with these regions. Then, we tested and validated whether the tumors in these specific regions could act as an imaging prognostic factor.

## 2 Materials and methods

### 2.1 Patient selection and image processing

Patient MRI images were obtained from various collections within the Cancer Imaging Archive (TCIA) (https://www.cancerimagingarchive.net) (19), including TCGA-LGG (https://wiki.cancerimagingarchive.net/pages/viewpage.action?pageId=5309188) (19), LGG-1p19qDeletion (https://wiki.cancerimagingarchive.net/display/Public/LGG-1p19qDeletion) (20), and TCGA-GBM (https://wiki.cancerimagingarchive.net/pages/viewpage.action?pageId=1966258) (19). The inclusion criteria were as follows: (a) pathological confirmation of newly diagnosed IDH-wildtype GBM or IDH-mutant astrocytoma according to WHO CNS5 (2); (b) availability of full clinical information, including age, sex, KPS, and survival time; and (c) high-resolution, pre-treatment T2-weighted MRI data without obvious metal artifacts or motion artifacts. The tumor boundary was outlined manually by two experienced neurosurgeons via MRIcro software (https://people.cas.sc.edu/rorden/mricro/mricro.html). In cases where the tumor volume discrepancy was < 5%, the tumor masks were merged to create a new unified tumor mask for analysis. However, if the discrepancy exceeded 5%, the determination of the tumor border was made by another senior neuroradiologist. Finally, 111 patients with IDH-wildtype GBM and 78 patients with IDH-mutant astrocytoma from the TCIA database were enrolled in further analyses (Table 1). Figure 1 presents the patient selection flow diagram. All MRI images and tumor lesion masks were registered to the high-resolution (1 mm^3^ isotropic resolution) Montreal Neurological Institute (MNI) space (Montreal, Quebec, Canada) by using a normalizing algorithm provided by SPM8 (http://www.fil.ion.ucl.ac.uk/spm/software/spm8) (21). After normalization, we calculated the volume and centroid of each tumor lesion using AFNI software (http://afni.nimh.nih.gov/afni/). The centroid of a registered tumor region was determined as the position represented by the mean coordinates of all voxels within the masked region along three orthogonal directions. To identify the distribution of tumors, the computed tumor centroids were mapped onto a template brain (Supplementary Table 1). In the last part of this study, we conducted a survival analysis to validate our findings by selecting glioma patients who were treated at Harbin Medical University Cancer Hospital between January 2016 and December 2017. Each patient underwent telephone follow-up every 3 months until loss to follow-up or death. The inclusion criteria and data processing methods remained consistent with the earlier analysis (Figure 1). The validation set comprised a total of 72 IDH-wildtype GBM patients and 48 IDH-mutant astrocytoma patients who were enrolled in the study (patient characteristics are summarized in Supplementary Table 2). The parameters of T2 images acquired from our center were as follows: flip angle 8°, matrix 256 × 256, and slice thickness 5 mm. Written informed consent was obtained from all patients. This research was ethically approved by the Ethics Committee of Harbin Medical University (KY2021-42).

**Table 1:** General information of selected patients.

	**IDH-wildtype GBM**	**IDH-mutant astrocytoma**	***P*-value**
**Total number**	111 (100)	78 (100)	
**Sex**			
Male	61 (54.95)	37 (47.44)	0.308^a^
Female	50 (45.05)	41 (52.56)	
**Age (y)**			
≥50	86 (77.47)	32 (41.03)	< 0.001^a^
< 50	25 (22.52)	46 (58.97)	
Mean ± SD	58.30 ± 14.57	45.41 ± 13.52	< 0.001^b^
**KPS**			
≥80	85 (76.57)	67 (85.90)	0.112^a^
< 80	26 (23.42)	11 (14.10)	
Mean ± SD	79.19 ± 14.09	86.58 ± 12.39	< 0.001^b^
**Survival (y)**			
Mean ± SD	13.51 ± 11.92	39.71 ± 36.77	< 0.001^b^
**Tumor volume (cm** ^3^ **)**			
< 40	81 (72.97)	55 (70.51)	0.711^a^
≥40	30 (27.03)	23 (29.49)	
Mean ± SD	34.56 ± 30.88	41.64 ± 53.00	0.812^b^

Data are shown as the number of patients with percentages in parentheses or the mean ± SD.

^a^Results of the chi-square test.

^b^Results of the t-test.

### 2.2 VLSM analysis

To identify the brain regions that were strongly correlated with OS or KPS in each glioma subgroup, VLSM analysis was performed using MATLAB (R2015b). The general linear model (Y = βX + ε) was independently applied to each voxel. In this context, Y represents the presence (1) or absence (0) of glioma involvement in each patient, while X represents the clinical characteristic matrix (X1 = OS or KPS, X2 = patient age, X3 = patient sex, and X4 = glioma volume). The parameter β estimates the correlation between the voxel and clinical characteristic (OS and KPS in this study), whereas ε represents the residual. By employing the general linear model, voxels that exhibited a significant association with patient OS or KPS were identified while controlling for the influence of age, sex, and tumor volume. To further validate these findings, a permutation test (*n* = 500) was performed to correct for potential false positives (*P* < 0.05, FDR-corrected). The *t*-value of a voxel that exceeded the *t*-values observed in more than 95% of permutations was deemed statistically significant in this study and included in the results (14). ROI1 was defined as the brain region associated with lower KPS in GBM patients. Similarly, ROI2 was identified as the brain region linked to shorter OS in GBM patients. ROI3 represented the brain region associated with decreased KPS in astrocytoma patients.

### 2.3 Impairments of brain structure and function

To evaluate the impact of these specific brain regions on the brain structural connectome, we downloaded a white matter tractography atlas from NatBrain Lab (http://www.natbrainlab.com) and then calculated the overlapping volume and proportion between ROIs and each structure in the template. The final overlapping map was visualized by MRIcroGL (http://www.cabiatl.com/mricrogl/). NeuroSynth utilizes a combination of text mining, meta-analysis, and machine learning techniques to generate probabilistic mappings between cognitive and neural states. These mappings have broad applicability across various neuroimaging studies and analyses (22). To assess potential brain function impairment associated with these newly identified ROIs, we performed a meta-analytic functional decoding analysis using the NeuroSynth database (www.neurosynth.org) and NiMARE. Specifically, the Neurosynth ROI association method and feature terms downloaded from the 50 topic terms (v3; https://github.com/NeuroanatomyAndConnectivity/gradient_analysis/blob/master/gradient_data/neurosynth/v3-topics-50-keys.txt) were used. NiMARE software (https://github.com/neurostuff/NiMARE) was used to acquire image-based meta-analysis results. The final results of impaired brain psychological function are shown in Supplementary Tables 3–5. Higher scores of brain activity indicate a greater impact on cognitive function, which is visually represented in the word cloud by darker colors and larger sizes corresponding to the respective terms. These analyses aimed to identify the macrostructural disruption of white matter and cognitive dysfunction most strongly associated with these ROIs.

### 2.4 Survival analysis

To test whether the involvement of these ROIs could act as prognostic predictive factors, receiver operating characteristic (ROC) analysis was performed using the survival ROC package. Taking into account the average survival duration of glioblastoma, which ranges from 12 to 14 months, and our median follow-up time of around 3 years for astrocytoma, we designated 1-year survival status for glioblastoma patients and 3-year survival status for astrocytoma patients as the observation endpoints. The predictive performance is quantified using the area under the ROC curve (AUC), where an AUC of 1 indicates perfect prediction and an AUC of 0.5 indicates random prediction.

Next, patients in the IDH-wildtype GBM group were classified into the high-risk group if their tumor lesions overlapped with either one or both ROI1 (KPS-associated regions in GBM) and ROI2 (OS-associated regions in GBM). Conversely, patients were classified into the low-risk group if their tumor lesions did not overlap with either ROI1 or ROI2. Similarly, in IDH-mutant astrocytoma, patients were categorized as high-risk if their lesions overlapped with ROI3 (KPS-associated regions in astrocytoma) and as low-risk if there was no overlap with ROI3. OS was defined as the time from diagnosis to death. Survival analyses between the high-risk and low-risk groups were performed using Kaplan–Meier analyses, and the log-rank test was applied to assess the *P*-value.

### 2.5 Statistical analysis

In our dataset, the average age of patients was 52.98 years. The average tumor volume was 37.48 cm^3^. Therefore, we selected neighboring cutoffs of 50 years for age and 40 cm^3^ for tumor volume as grouping criteria for chi-square distribution tests. Regarding the KPS score, patients with a score of 80 or above are generally considered able to live and work independently without special care. Hence, we used a cutoff of 80 for the statistical analysis grouping criterion. The chi-squared test was used to compare sex, age (< 50 y vs. ≥50 y), KPS (< 80 vs. ≥80), tumor volume (< 40 vs. ≥40 cm^3^), and spatial location distributions. A *t*-test was applied to examine differences in age, KPS survival time, and tumor volume. A *P*-value of < 0.05 was considered to be statistically significant. The baseline characteristics of patients from the TCIA are listed in Table 1.

## 3 Results

### 3.1 Patient characteristics

A total of 111 patients (61 male and 50 female subjects) with IDH-wildtype GBM and 78 patients (31 male and 47 female subjects) with IDH-mutant astrocytoma who met the inclusion criteria were enrolled in the study. Compared with astrocytoma patients, GBM patients were older (58.30 ± 14.57 vs. 45.41 ± 13.52, *P* < 0.001), had a lower KPS (79.19 ± 14.09 vs. 86.58 ± 12.39, *P* < 0.001), and had a shorter OS (13.51 ± 11.92 vs. 39.71 ± 36.77, *P* < 0.001). No significant difference in tumor volume was found between the two cohorts. The baseline characteristics of these patients from the TCIA are summarized in Table 1. IDH-wildtype GBM and IDH-mutant astrocytoma did not exhibit significant differences in their spatial distribution across various brain regions (*P* = 0.091). However, astrocytoma demonstrated a significantly higher proportion of distribution in the frontal lobe compared to GBM (21.62 vs. 42.31%, Supplementary Table 1).

### 3.2 VLSM identified regions

**Figure 2:**
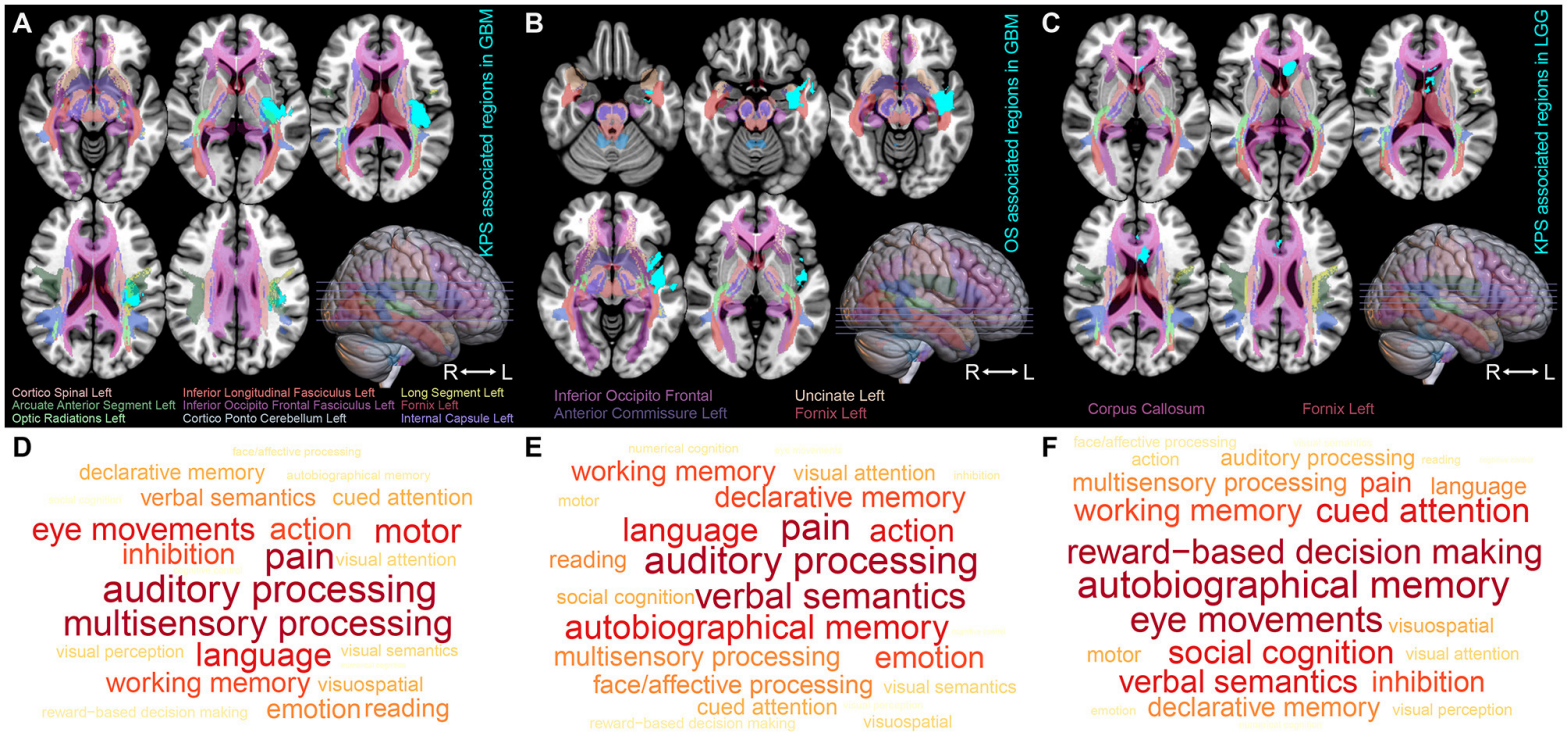
ROI-induced structure and function impairment. **(A–C)** Overlapping map of Natbrainlab brain structures and ROIs. VLSM was performed to identify the specific regions that are correlated with OS or KPS. Only significant voxels (permutation testing, *n* = 500, *P* < 0.01) are rendered in cyan. IDH-wildtype GBM KPS-associated regions, IDH-wildtype GBM OS-associated regions, and IDH-mutant astrocytoma KPS-associated regions are defined as ROI1, ROI2, and ROI3, respectively. **(D–F)** Brain neurocognitive dysfunction induced by ROI1, ROI2, and ROI3. The size of the word corresponds to the cognitive function distribution.

Using VLSM analysis, we identified the brain regions that were associated with KPS and survival time. The regions correlated with low KPS scores in IDH-wildtype GBM were primarily located in the insular cortex, planum polare, parahippocampal gyrus, planum temporale, central opercular cortex, and parietal operculum cortex of the left hemisphere (ROI1, *t*-value = 2.38, Figure 2A). The regions that were significantly associated with short OS in GBM included the left parahippocampal gyrus, planum polare, temporal pole, middle temporal gyrus, superior temporal gyrus, and central opercular cortex (ROI2, *t*-value = 1.66, Figure 2B). Tumors occurring at the subcallosal cortex and cingulate gyrus tended to lead to a lower KPS score in IDH-mutant astrocytoma (ROI3, *t*-value = 2.36, Figure 2C). However, no significant result was found in terms of OS-associated regions in IDH-mutant astrocytoma.

### 3.3 Structure and cognitive functions associated with ROIs

To investigate the impact of these ROIs on the brain structure, we started by comparing the spatial overlap between ROIs and the normal brain white matter structure (NatBrainLab white matter tractography template) (Table 2). We found that ROI1 overlapped with the corticospinal tract, inferior longitudinal fasciculus (ILF), inferior occipitofrontal fasciculus (IOFF), fornix, internal capsule, cortico-ponto-cerebellar (CPC) pathway, and optic radiations (Figure 2A). The main structures that overlapped with ROI2 included the IOFF, fornix, uncinate fasciculus, and anterior commissure (Figure 2B). The corpus callosum and fornix were the structures that were included in ROI3 (Figure 2C). Next, we conducted meta-analytic functional decoding of the three ROIs among 50 topics from NeuroSynth. The results of significant psychological terms are summarized in Supplementary Tables 3–5. In ROI1, the most associated functions were multisensory processing, auditory processing, pain, language, motor, and eye movements (Figure 2D). For ROI2, the related brain functions included verbal semantics, auditory processing, pain, autobiographical memory, language, and action (Figure 2E). The brain function that showed the highest correlation with ROI3 was reward-based decision-making, followed by autobiographical memory, eye movements, cued attention, social cognition, verbal semantics, and working memory (Figure 2F).

**Table 2:** ROI-induced brain structure impairment.

**ROI**	**Structure name**	**Overlapping volume**	**Structure volume**	**Overlapping percentage**
GBM KPS-associated regions	Cortico spinal left	827	27,876	0.02
	Inferior longitudinal fasciculus left	299	13,859	0.02
	Inferior occipito frontal fasciculus left	313	11,032	0.02
	Fornix left	504	10,145	0.04
	Internal capsule left	608	10,078	0.06
	Cortico ponto cerebellum left	271	6,672	0.04
	Optic radiations left	1,288	4,419	0.29
	Arcuate anterior segment left	1,402	4,095	0.34
	Long segment left	916	2,984	0.30
GBM OS-associated regions	Inferior occipito frontal fasciculus left	610	11,032	0.05
	Fornix left	287	10,145	0.02
	Uncinate left	496	7,310	0.06
	Anterior commissure left	120	4,875	0.02
Astrocytoma KPS-associated regions	Corpus callosum left	1,794	46,399	0.03
	Fornix left	212	10,145	0.02

ROI, Region of Interest. Volume refers to the voxel number.

### 3.4 Survival analysis

**Figure 3:**
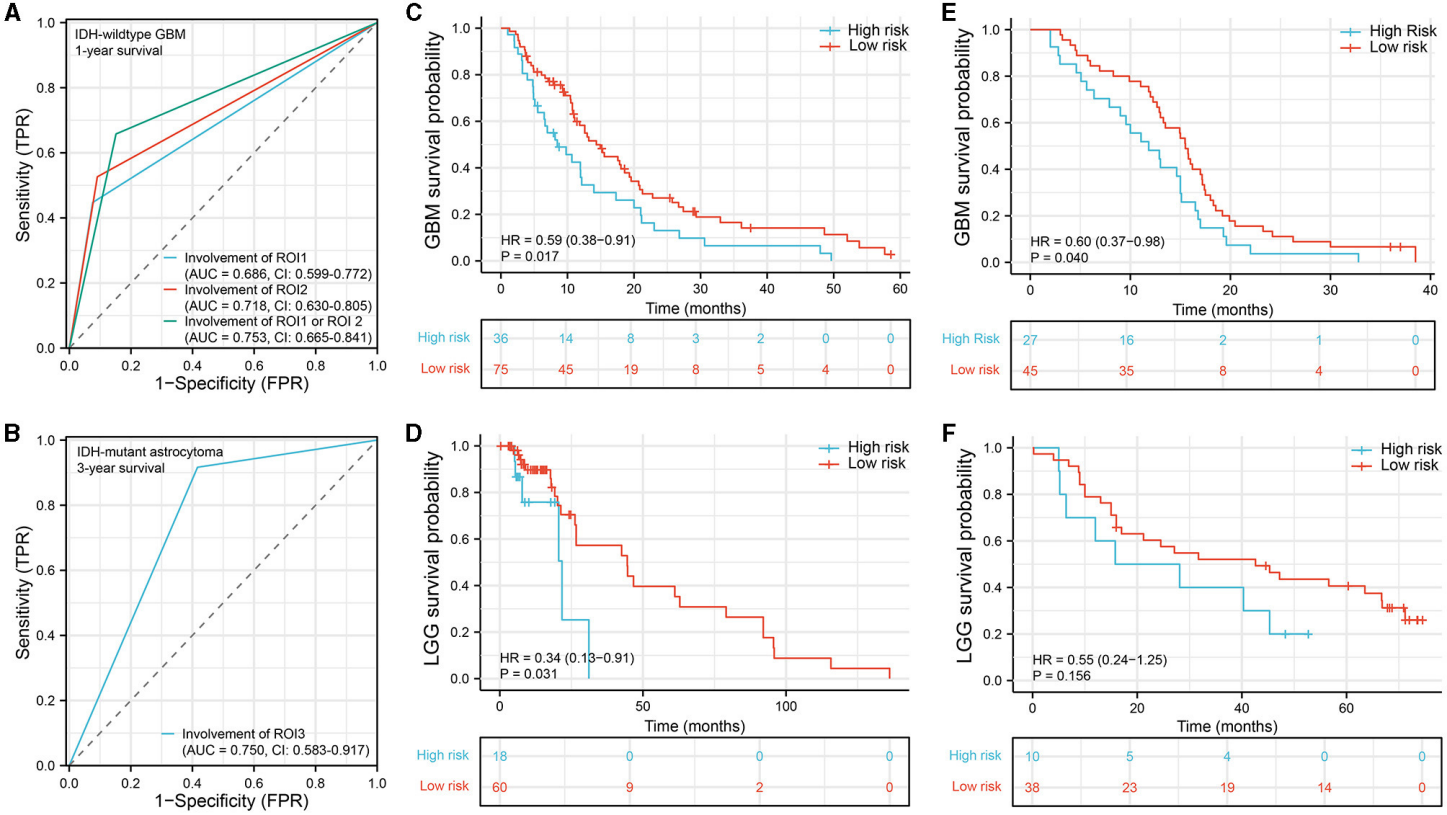
Survival analysis. **(A, B)** ROC curve analysis to predict 1-year survival status in IDH-wildtype GBM and 3-year survival status in IDH-mutant astrocytoma. **(C–F)** Kaplan–Meier survival curve analysis was performed to compare the survival between the high-risk group and the low-risk group of patients in the IDH-wildtype GBM cohort **(C)**, IDH-mutant astrocytoma cohort **(D)**, IDH-wildtype GBM validation cohort **(E)**, and IDH-mutant astrocytoma validation cohort **(F)**. ROC, receiver operating characteristic; AUC, area under the ROC curve.

To evaluate the prognostic diagnostic capability of the ROIs, we conducted an ROC curve analysis. In GBM, the combined predictive ability of ROI1 and ROI2 for patient survival status at 1-year was stronger than the individual predictive abilities of each ROI (AUC = 0.753, CI: 0.665–0.841, Figure 3A). In astrocytoma, the involvement of ROI3 also exhibited a certain predictive capacity in assessing patient survival status at 3 years (AUC = 0.750, CI: 0.583–0.917, Figure 3B). Next, we classified all patients into a high-risk group and a low-risk group according to their tumor location. In the IDH-wildtype GBM and IDH-mutant astrocytoma cohorts, patients whose tumor lesions completely or partially overlapped with one of the three ROIs (ROI1 and ROI2 in GBM, ROI3 in astrocytoma) were enrolled in the high-risk group. Otherwise, patients were included in the low-risk group. As expected, the survival curves were significantly different in both IDH-wildtype GBM (Figure 3C, *P* = 0.017, HR = 0.59, 95% CI = 0.38–0.91) and IDH-mutant astrocytoma (Figure 3D, *P* = 0.031, HR = 0.34, 95% CI = 0.13–0.91), suggesting that damage to these VLSM-identified regions tended to lead to a poor outcome. To validate our findings, we selected 72 IDH-wildtype GBM patients and 48 IDH-mutant astrocytoma patients who met the same inclusion criteria from our center and divided them into two groups using the same procedure. Similarly, there was a significant difference in overall survival in the GBM cohort (Figure 3E, *P* = 0.040, HR = 060, 95% CI = 0.37–0.98). However, in IDH-mutant astrocytoma patients, we only found a non-significant tendency toward poor survival in the high-risk group (Figure 3F, *P* = 0.156, HR = 0.55, 95% CI = 0.24–1.25).

## 4 Discussion

In the present research, we identified the specific regions that were correlated with low KPS scores or short OS in IDH-wildtype GBM (*n* = 111) and IDH-mutant astrocytoma (*n* = 78). We found that the VLSM-identified brain areas in IDH-wildtype GBM mainly overlapped with IOFF, ILF, fornix, and corticospinal tract and were associated with neurocognitive functions, including auditory processing, multisensory processing, verbal semantics, language, and pain. Unlike GBM, the region that was associated with KPS in IDH-mutant astrocytoma overlapped with the corpus callosum and fornix. Moreover, it appeared that KPS-associated regions in IDH-mutant astrocytoma were more likely to relate to higher brain functions, such as autobiographical memory, reward-based decision-making, cued attention, and social cognition. More importantly, our results suggested that invasion of these specific regions heralded a poor prognosis in both IDH-wildtype GBM and IDH-mutant astrocytoma. Our validation set confirmed the generalizability and applicability of the findings in IDH-wildtype GBM.

### 4.1 Brain regions correlated with OS or KPS

A previous study demonstrated that the spatial distribution of GBM with short OS was significantly different from that of GBM with long survival (9). Tumors located in the left frontotemporal region are more likely to induce a poor prognosis, which is corroborated by our results in IDH-wildtype GBM. In our survival analysis, the involvement of the left parahippocampal gyrus, temporal pole, middle temporal gyrus, and superior temporal gyrus indicated poor OS. This conclusion held true for the IDH-wildtype GBM patients from both the TCIA database (Figure 3A) and our center (Figure 3C). For KPS, Marina et al. (23) reported that impaired KPS of GBM patients was most commonly seen with the corpus callosum or thalamic involvement, which were adjacent to the KPS-associated areas in our results (Figure 2A). In another study, Chaichana et al. (24) found that a midline location of a GBM lesion was less likely to induce a low KPS status at diagnosis. Similarly, our VLSM-identified regions were also far from the midline (Figure 2A). In contrast, limited information is available about IDH-mutant astrocytoma regarding how lesion location affects KPS. Our VLSM analysis showed that, unlike GBM, regions (partial subcallosal cortex and cingulate gyrus) that were strongly associated with low KPS in IDH-mutant astrocytoma were near the midline, suggesting that IDH-wildtype GBM and IDH-mutant astrocytoma had distinct KPS impairment patterns. Moreover, IDH-mutant astrocytoma invasion into these regions led to a shorter OS in TCIA patients (*P* = 0.031, Figure 3D). However, the validation result was not sufficiently significant to verify this finding (*P* = 0.156, Figure 3F).

### 4.2 Cognitive dysfunction in glioma

Deficits in cognitive functioning are common among patients with glioma (25). A meta-analysis indicated that the majority of glioma patients had an impairment in at least one neurocognitive domain (memory, language, learning, attention, perceptual-motor function, and executive function) (26). Relative factors that may impact neurocognitive impairment include the tumor (e.g., tumor size, tumor location, pathological grade, and molecular alterations), the patient (e.g., age and education level), and the treatment modalities (chemotherapy, radiotherapy, and antiepileptic drugs) (26, 27). Among all cognitive dysfunctions induced by a brain tumor, memory, attention, and executive functioning have the highest incidence rate (29–90%) (28, 29), which is in concordance with our results showing close associations with memory and attention in both IDH-wildtype GBM and IDH-mutant astrocytoma.

In this study, we divided all glioma patients into the IDH-wildtype GBM cohort and IDH-mutant astrocytoma cohort according to their pathological subtypes, which could also determine the severity of brain dysfunction. Neurocognitive deficits are often considered to be more relevant in patients with LGG (astrocytoma and oligodendroglioma), given their younger age and much longer survival time (30). With the same rationale, there is a bias toward testing cognition in LGG patients but not in GBM patients (31). The limited analyses for tumor grade subgroups suggested that patients with GBM exhibited more severe disruptions in verbal learning, language, processing speed, and executive functioning (30, 32, 33). Similarly, our VLSM-identified regions in IDH-wildtype GBM were also related to more obvious language and processing speed impairment (Figures 2D, E). In addition, several reports demonstrated that patients with GBM tended to suffer from more severe and frequent cognitive deficits (32, 34, 35). One theory to explain these different cognitive and neurological symptom burdens is the tumor growth rate (36). Because of the characteristics of a slower-growing pattern, there is more time for LGG patients to integrate the glioma-neural network, which may eventually lead to neural functional reorganization and cognitive rehabilitation (27).

With the advancement of research on brain functional and structural networks, we have come to realize that different regions of the brain are closely interconnected in terms of both function and structure (37). Damage to a specific brain region can lead to widespread functional impairments throughout the brain. Therefore, in personalized treatment for gliomas, techniques such as functional magnetic resonance imaging (fMRI) can be utilized to delineate the functional boundaries of tumors, reducing the occurrence of postoperative complications (38). Our study, conducted at a population level, has provided initial insights into the differences in functional impairments between glioblastoma and astrocytoma, thus complementing the existing research in this field. In future glioma treatments, apart from improving patient survival, it is equally important to preserve normal brain function to the greatest extent possible.

### 4.3 Alterations of white matter integrity

White matter fiber tracts are responsible for facilitating communication between networks of distributed brain regions (39). Therefore, the maintenance of white matter integrity is critically important for cognitive performance, and a positive correlation has been established between cognitive performance and white matter integrity (40). During the process of tumor invasion, glioma cells preferably migrate along the white matter fibers, leading to structural alteration of the surrounding tissues (41). Reduced integrity of white matter tracts has been proven to be associated with deficient cognitive function in glioma (42, 43). It cannot be ignored that patients with astrocytoma have a life expectancy of 5–15 years. Therefore, they may experience white matter remodeling and cognitive rehabilitation, which means that their neurocognitive performance is dynamically changing (44). Moreover, a more extensive glioma invasion in white matter fibers always indicates a higher tumor recurrence rate and poorer prognosis (45, 46). Similarly, we found that the involvement of white matter tracts in GBM ROIs was also more severe than that in IDH-mutant astrocytoma ROIs. White matter fiber tracts form the basis for functional interactions between brain regions (47). In our results, the ROI-induced cognitive dysfunction corresponded clinically with the function of the damaged white matter (48).

In our previous studies, we have already identified distinct spatial distribution patterns among different subtypes of gliomas (49). In this study, we further discovered that the underlying mechanisms contributing to the clinical functional impairments caused by GBM and astrocytoma differ from each other. Previously, certain traditional anatomical structures such as the brainstem, corpus callosum, and thalamus have been considered as prognostic factors for poor outcomes (50). However, in this study, we went beyond anatomical boundaries and employed the VLSM method to identify ROIs that can also serve as prognostic indicators. This breakthrough allows neurosurgeons to roughly assess patient prognosis before surgery based on the overlap of tumor boundaries with these regions of interest. If a patient's region of interest in the brain is invaded by a tumor, it indicates a higher risk of poor prognosis. Therefore, more frequent follow-up examinations and more aggressive treatments may potentially benefit the patient. Moving forward, we aim to integrate these novel imaging diagnostic features with clinical characteristics, genomics, and other factors to enhance the accuracy of survival prediction in glioma patients (16).

### 4.4 Limitations

This research has several limitations worth noting. During the data collection process, due to the limited number of IDH-mutant and 1p/19q-codeleted oligodendroglioma cases, they were not included in this study. In future, we hope to supplement this aspect by obtaining a larger sample size and multicenter data. This will also allow us to further validate the conclusions we have drawn in IDH-wildtype GBM and IDH-mutant astrocytoma. Second, no OS-associated regions were found in the IDH-mutant astrocytoma cohort, which requires further confirmation. Among the patients in the IDH-mutant astrocytoma validation set, there was a non-significant trend toward a survival benefit among the patients in the low-risk groups. Further studies based on large-scale populations are needed to validate whether the involvement of the specific regions is a predictive indicator in IDH-mutant astrocytoma. The tumor MRI data in our study were collected from multiple centers, and variations in acquisition parameters may have an impact on the accurate delineation of tumor boundaries. This is a common challenge faced by studies of a similar nature. In future, it is important to prioritize the acquisition of high-quality, high-resolution data to minimize such issues.

## 5 Conclusions

Our study has identified specific brain regions that showed correlations with KPS or OS in IDH-wildtype GBM and IDH-mutant astrocytoma. These regions may be associated with structural variations and neurocognitive dysfunction. Importantly, we found that the involvement of these regions, particularly in IDH-wildtype GBM, was indicative of poorer survival. The findings from our study provide novel insights into the distinct biological behaviors and clinical outcomes observed in IDH-wildtype GBM and IDH-mutant astrocytoma. Furthermore, the neuroimaging biomarkers discovered in this study have the potential to aid neurosurgeons in gaining a deeper understanding of the underlying pathogenic mechanisms of glioma and assessing the prognosis of glioma patients.

## Data availability statement

The raw data supporting the conclusions of this article will be made available by the authors, without undue reservation.

## Ethics statement

The studies involving humans were approved by the Ethics Committee of Harbin Medical University. The studies were conducted in accordance with the local legislation and institutional requirements. Written informed consent for participation was not required from the participants or the participants' legal guardians/next of kin in accordance with the national legislation and institutional requirements.

## Author contributions

HB: Conceptualization, Data curation, Formal analysis, Funding acquisition, Methodology, Resources, Visualization, Writing—original draft, Writing—review & editing. HW: Data curation, Investigation, Methodology, Resources, Software, Writing—review & editing. QS: Data curation, Investigation, Methodology, Resources, Software, Writing—review & editing. YW: Data curation, Methodology, Resources, Software, Writing—review & editing. HL: Data curation, Methodology, Resources, Software, Writing—review & editing. PL: Conceptualization, Supervision, Validation, Writing—review & editing. ZL: Conceptualization, Funding acquisition, Supervision, Validation, Writing—review & editing.
