# Supplementary material for: The involvement of brain regions associated with lower KPS and shorter survival time predicts a poor prognosis in glioma
Source: Front Neurol. 2023 Dec 4;14:1264322. doi: 10.3389/fneur.2023.1264322 (PMC10725945; doi:10.3389/fneur.2023.1264322)
Supplement: Supplementary file 1 [file Data_Sheet_1.docx]

Supplementary Material

# Supplementary Tables

**Supplementary Table 1.** Spatial distribution of IDH-wildtype GBM and IDH-mutant astrocytoma

| **Location** | **IDH-wildtype GBM**  **111 (100)** | **IDH-mutant astrocytoma**  **78 (100)** |
| --- | --- | --- |
| Frontal lobe | 24 (21.62) | 33 (42.31) |
| Parietal lobe | 18 (16.22) | 10 (12.82) |
| Temporal lobe | 22 (19.82) | 13 (16.67) |
| Occipital lobe | 13 (11.71) | 6 (7.69) |
| Insula lobe | 10 (9.01) | 6 (7.69) |
| Subcortex | 22 (19.82) | 7 (8.97) |
| Brainstem | 1 (0.90) | 2 (2.56) |
| Cerebellum | 1 (0.90) | 1 (1.28) |

Data are shown as the number of patients with percentages in parentheses

*P* = 0.091

**Supplementary Table 2.** General information of selected patients in the validation set

|  | **IDH-wildtype GBM** | **IDH-mutant astrocytoma** | ***P* value** |
| --- | --- | --- | --- |
| **Total Number** | 72 (100) | 48 (100) |  |
| **Sex** |  |  |  |
| Male | 44 (61.11) | 27 (56.25) | 0.596^a^ |
| Female | 28 (38.89) | 21 (43.75) |  |
| **Age (y)** |  |  |  |
| ≥ 50 | 57 (79.17) | 20 (41.67) | <0.001^a^ |
| <50 | 15 (20.83) | 28 (58.33) |  |
| Mean ± SD | 59.97 ± 12.43 | 43.56 ± 16.04 | <0.001^b^ |
| **KPS** |  |  |  |
| ≥ 80 | 57 (79.17) | 39 (81.25) | 0.780^a^ |
| < 80 | 15 (20.83) | 9 (18.75) |  |
| Mean ± SD | 80.83 ± 13.40 | 85.21 ± 13.21 | 0.042^b^ |
| **Survival (y)** |  |  |  |
| Mean ± SD | 14.84 ± 10.03 | 36.14 ± 25.64 | <0.001^b^ |
| **Tumor Volume (cm^3^)** |  |  |  |
| <40 | 49 (68.06) | 30 (62.50) | 0.530^a^ |
| ≥40 | 23 (31.94) | 18 (37.50) |  |
| Mean ± SD | 39.42 ± 25.27 | 45.52 ± 50.61 | 0.332^b^ |

Data are shown as the number of patients with percentages in parentheses or the mean ± SD.

^a^Results of the chi-square test.

^b^Results of t test.

**Supplementary Table 3.** Functional decoding results of ROI1

| **Term** | **pForward** | **pReverse** | **Activity score** |
| --- | --- | --- | --- |
| auditory processing | 1.24E-10 | 2.37E-22 | 0.634829472 |
| pain | 3.95E-32 | 1.18E-17 | 0.631626899 |
| multisensory processing | 0.123799894 | 0.04168943 | 0.526056546 |
| motor | 2.29E-51 | 0.171020032 | 0.516165922 |
| language | 4.61E-10 | 0.681056012 | 0.505951099 |
| eye movements | 7.42E-233 | 0.963518105 | 0.501143924 |
| action | 1.92E-31 | 0.929981447 | 0.498552607 |
| inhibition | 0.056601522 | 0.309665288 | 0.486374526 |
| working memory | 2.11E-16 | 0.187702549 | 0.48006444 |
| emotion | 1.29E-205 | 0.115045581 | 0.479826609 |
| reading | 4.76E-201 | 0.0536597 | 0.475411157 |
| verbal semantics | 3.26E-89 | 0.223590121 | 0.474345976 |
| cued attention | 4.07E-20 | 0.078236179 | 0.472722507 |
| declarative memory | 3.01E-06 | 0.026560734 | 0.472529037 |
| visuospatial | 3.08E-12 | 0.025035111 | 0.467042297 |
| visual attention | 1.54E-165 | 0.005225751 | 0.465484828 |
| visual perception | 3.54E-58 | 0.036851936 | 0.460514021 |
| visual semantics | 2.93E-27 | 0.010144593 | 0.458455144 |
| reward-based decision making | 1.29E-11 | 0.001097245 | 0.452279382 |
| autobiographical memory | 1.24E-136 | 0.023515738 | 0.445102178 |
| face/affective processing | 2.33E-16 | 0.000155917 | 0.442950681 |
| social cognition | 0.000369537 | 8.29E-06 | 0.438972269 |
| cognitive control | 0 | 0.768473595 | 0.432848589 |
| numerical cognition | 6.05E-262 | 0.000427742 | 0.396366395 |

pForward: The probability of observing activation in specific brain regions given the presence a particular term (P (Activation | Term), or ‘forward inference’); pReverse: The probability of a term occurring in an article given the presence of activation in a particular brain region (i.e., P (Term | Activation), or reverse inference).

**Supplementary Table 4.** Functional decoding results of ROI2

| **Term** | **pForward** | **pReverse** | **Activity score** |
| --- | --- | --- | --- |
| auditory processing | 1.30E-12 | 1.10E-05 | 0.58332414 |
| pain | 1.20E-26 | 0.000437413 | 0.575281069 |
| verbal semantics | 1.59E-35 | 0.029389616 | 0.550564785 |
| autobiographical memory | 8.34E-50 | 0.08365761 | 0.54434619 |
| language | 1.20E-05 | 0.403706279 | 0.51494947 |
| action | 2.20E-18 | 0.895966179 | 0.502676515 |
| emotion | 5.85E-148 | 0.696828858 | 0.493692708 |
| declarative memory | 2.47E-07 | 0.679966703 | 0.493682478 |
| working memory | 2.30E-09 | 0.55412298 | 0.488927988 |
| multisensory processing | 0.483930945 | 0.39439777 | 0.485899416 |
| face/affective processing | 3.07E-06 | 0.372347643 | 0.483897811 |
| reading | 5.31E-140 | 0.230186504 | 0.480802023 |
| cued attention | 9.44E-13 | 0.138387809 | 0.471263739 |
| visual attention | 1.14E-114 | 0.043356591 | 0.468720069 |
| social cognition | 0.188057845 | 0.05222637 | 0.467544284 |
| visual semantics | 6.78E-16 | 0.087916885 | 0.465843645 |
| visuospatial | 9.86E-09 | 0.025282852 | 0.458504092 |
| motor | 7.94E-19 | 0.005249802 | 0.458496128 |
| reward-based decision making | 3.68E-07 | 0.01513339 | 0.455774011 |
| numerical cognition | 1.90E-88 | 0.079570079 | 0.439691642 |
| inhibition | 0.000146423 | 0.000203011 | 0.43485155 |
| visual perception | 1.28E-38 | 0.002351984 | 0.424410811 |
| eye movements | 7.17E-95 | 0.008954426 | 0.404318255 |
| cognitive control | 0 | 0.478064233 | 0.34073583 |

pForward: The probability of observing activation in specific brain regions given the presence a particular term (P (Activation | Term), or ‘forward inference’); pReverse: The probability of a term occurring in an article given the presence of activation in a particular brain region (i.e., P (Term | Activation), or reverse inference).

**Supplementary Table 5.** Functional decoding results of ROI3

| **Term** | **pForward** | **pReverse** | **Activity score** |
| --- | --- | --- | --- |
| autobiographical memory | 3.61E-09 | 0.015239111 | 0.604785463 |
| reward-based decision making | 0.262094448 | 0.001097933 | 0.596536771 |
| eye movements | 1.22E-13 | 0.248557463 | 0.561070612 |
| cued attention | 0.016846004 | 0.426462212 | 0.527262819 |
| social cognition | 0.313242599 | 0.422560053 | 0.524213814 |
| verbal semantics | 1.58E-10 | 0.863502536 | 0.508125881 |
| working memory | 0.00658027 | 0.978462195 | 0.500943278 |
| inhibition | 0.7641165 | 0.923292171 | 0.496972276 |
| pain | 3.92E-10 | 0.87758395 | 0.492824815 |
| declarative memory | 0.00608256 | 0.684770062 | 0.488192141 |
| multisensory processing | 0.461677334 | 0.295986029 | 0.466693978 |
| language | 0.00109972 | 0.312556673 | 0.463661547 |
| auditory processing | 1.77E-08 | 0.31424566 | 0.456267513 |
| motor | 4.67E-06 | 0.064283437 | 0.447825648 |
| visuospatial | 0.001638383 | 0.124705446 | 0.445308668 |
| action | 1.29E-07 | 0.189352745 | 0.44502217 |
| visual attention | 1.31E-31 | 0.042677796 | 0.441757959 |
| visual perception | 5.43E-10 | 0.177512826 | 0.437669649 |
| face/affective processing | 0.000757031 | 0.069886781 | 0.434714696 |
| emotion | 1.39E-35 | 0.019638739 | 0.432192528 |
| reading | 5.34E-35 | 0.015235753 | 0.4296736 |
| visual semantics | 1.47E-06 | 0.050908434 | 0.422026434 |
| numerical cognition | 1.39E-18 | 0.065702828 | 0.366043159 |
| cognitive control | 6.18E-127 | 0.029733518 | 0.134464752 |

pForward: The probability of observing activation in specific brain regions given the presence a particular term (P (Activation | Term), or ‘forward inference’); pReverse: The probability of a term occurring in an article given the presence of activation in a particular brain region (i.e., P (Term | Activation), or reverse inference).
